# Biosynthesis of cyanophycin by high-cell-density cultivation of recombinant *Escherichia coli* for corrosion-resistant biomaterial applications

**DOI:** 10.1007/s00449-026-03412-9

**Published:** 2026-08-21

**Authors:** Saroj Raj Kafle, Anirudh Mukunth, Arum Han, Arul Jayaraman, Aristos Aristidou

**Affiliations:** Artie McFerrin Department of Chemical Engineering, Texas A&M University, College Station, TX 77840 USA

**Keywords:** Cyanophycin (CGP), High cell density, *Escherichia coli*, Corrosion inhibition

## Abstract

Cyanophycin granule polypeptide (CGP), also known as multi-l-arginyl-poly(aspartic acid), is a biodegradable biopolymer composed primarily of aspartic acid and arginine. Due to its versatile functional properties, CGP has attracted increasing interest for potential applications in food, medicine, cosmetics, agriculture, and corrosion inhibition. The objective of this study was to biosynthesize a biologically derived corrosion-inhibiting biomaterial using recombinant *Escherichia coli* BL21(DE3) expressing cyanophycin synthetase (CphA), followed by cost-effective induction strategy. Specifically, this work aimed to establish a high-cell-density cultivation process capable of achieving improved CGP production while reducing dependence on costly inducers such as IPTG. Initial shake-flask experiments demonstrated that lactose induction resulted in higher CGP production and biomass formation compared to IPTG induction. In addition, supplementation with phosphate, ribose, trace elements, yeast extract, and tryptone further improved CGP accumulation. Based on these findings, a high-cell-density fed-batch fermentation strategy using lactose as both inducer and carbon source was developed, maximum gravimetrically recovered crude soluble and insoluble CGP-containing fractions of 35.4 and 17.8 g/L. Product characterization was further supported by FTIR, XRD, MALDI-MS. Furthermore, the recovered CGP-related material was evaluated in preliminary corrosion inhibition experiments under acidic conditions and showed significant reduction in corrosion rates compared with untreated control samples, indicating its potential for future corrosion-protection applications.

**Graphical abstract:**

**Figure:**
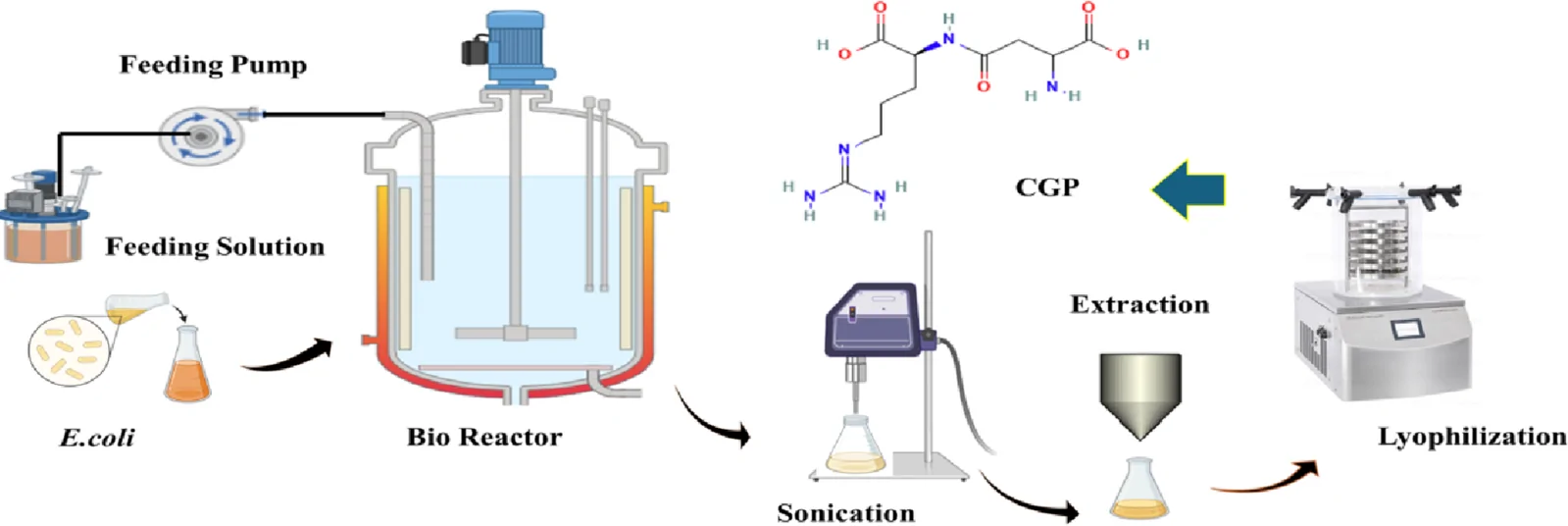

**Supplementary Information:**

The online version contains supplementary material available at https://doi.org/10.1007/s00449-026-03412-9.

## Introduction

Corrosion presents significant challenges in process industries such as oil, gas, and refining, with annual losses in the oil sector alone reaching billions of dollars. Globally, steel corrosion is estimated to cause economic damage exceeding $700 billion each year [1–3]. The corrosion inhibitor market is projected to grow substantially, reaching a value of $13.34 billion by 2034, driven by increasing demand for effective protection solutions [4].

Despite the availability of various corrosion mitigation strategies, there remains a pressing need for cost-effective and environmentally sustainable inhibitors [5–7]. Among emerging approaches, microbial corrosion inhibition stands out for its efficiency and eco-friendliness. Many microorganisms are easy to cultivate and pose minimal risks to human health and the environment, positioning microbial methods as a promising alternative to conventional chemical inhibitors [8, 9].

Recently, CGP has gained interest as a sustainable microbial corrosion inhibitor. It can be synthesized by various microorganisms such as cyanobacteria, *E. coli, Clostridium,* and *Pseudomonas* [10]. However, *E. coli* is the most commonly used host. The objective of this study was to biosynthesize a biologically derived corrosion-inhibiting biomaterial using an recombinant *E.coli* strain followed by cost-effective induction strategy. Specifically, this work aimed to establish a process capable of achieving high production rates, titers, and conversion yields, while eliminating the use of costly inducers such as IPTG for pathway protein overexpression. The approach also emphasizes versatility in producing diverse bioproducts [11, 12]. Recombinant *E.coli* strains are known to synthesize a high fraction of CGP in both soluble and insoluble forms [13, 14], with production levels strongly influenced by gene expression and medium composition. While significant progress has been made in the upstream biosynthesis of CGP using recombinant microbial systems, downstream recovery and purification remain important challenges for large-scale implementation. Since CGP is accumulated intracellularly, efficient cell disruption, separation of soluble and insoluble CGP fractions, removal of cellular impurities, and maintenance of product recovery during large-scale processing increase downstream processing complexity.

**Fig. 1:**
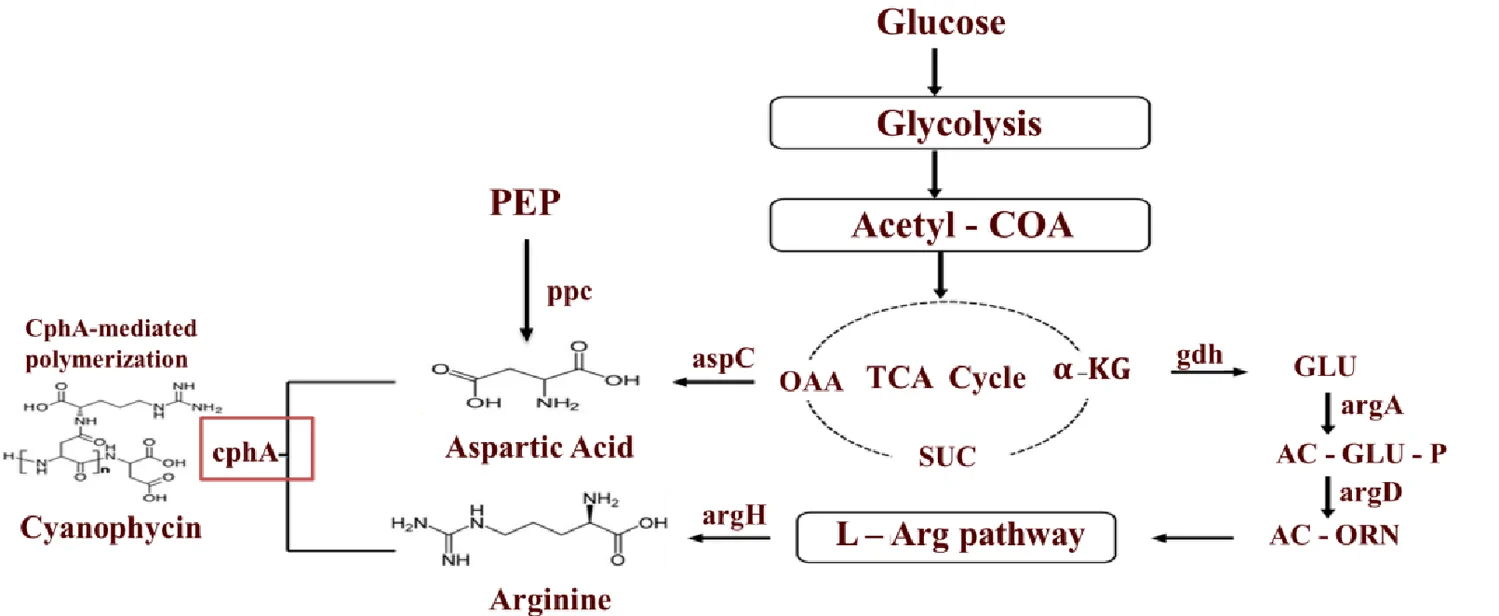
Proposed biosynthetic pathway for CGP production from glucose

In the present study, recombinant *E. coli* BL21 (DE3) carrying the pETDuet-1 plasmid encoding cyanophycin synthetase (CphA) from *Synecocystis* sp. PCC 6803 was employed for CGP biosynthesis. The recombinant construct enabled heterologous overexpression of CphA under the control of the T7 promoter system. During CGP biosynthesis, glucose is metabolized through central carbon metabolism including glycolysis and the TCA cycle to generate precursor metabolites such as oxaloacetate (OAA), aspartic acid, and arginine, which are subsequently polymerized by CphA to form CGP. A simplified biosynthetic pathway of CGP production is illustrated in Fig. 1.

Productivity in microbial systems is a function of cell density. Substrate limitation is one of the major constraints in batch fermentation. Various upstream and downstream strategies have been developed to intensify and optimize production, thereby enhancing both cell density and intracellular CGP production. Fed-batch fermentation enables a consistent supply of nutrients through strategies such as linear, exponential, pH–stat, DO-stat thereby enhancing titers, yield, and productivity of the system [15].

Until now, limited studies have investigated combined upstream cultivation and downstream recovery strategies for high-cell-density CGP production using fed-batch fermentation systems. In addition, several previously reported studies have relied on expensive conventional inducers, relatively low-cell-density cultivation, complex media formulations, or multistep downstream recovery procedures. To address these limitations, a recombinant *E.coli* BL21(DE3) strain was employed for enhanced CGP production using renewable carbohydrate feedstocks and a cost-effective lactose-based induction strategy. In this study, shake-flask experiments were initially performed to evaluate alternative low-cost inducing agents, followed by batch and fed-batch fermentation studies using phosphate, ribose, trace element, yeast extract, and tryptone supplementation to improve CGP production and biomass accumulation. The recovered CGP-related material obtained through an adapted recovery process was further evaluated in preliminary corrosion inhibition experiments under acidic conditions, where reduced corrosion behaviour compared with untreated controls was observed. Product characterization was additionally supported by FTIR, XRD, and MALDI-MS analyses.

## Materials and methods

### Chemicals and reagents

LB (Luria–Bertani) media for shake flask study was purchased from Becton, Dickinson and company sparks, USA. Yeast Extract (Research Product International, USA), D-glucose (VWR Chemicals, USA), magnesium sulphate (Thermofisher scientific, USA), citric acid (Thermofisher scientific, USA), potassium dihydrogen phosphate (Research Product International, USA), di-ammonium hydrogen phosphate (Avantor, USA) were used as the minimal media for fermentation process. Lactose (Thermofisher scientific, USA), D-Ribose (Thermofisher scientific, USA), Organic Phosphate (Thermofisher scientific, USA) were used as inducing agents and addition nutrients. Ammonium hydroxide (Thermofisher scientific, USA), sodium hydroxide (Thermofisher scientific, USA) and hydrochloric acid (Thermofisher scientific, USA) were used for pH adjustment. Tryptone (Thermofisher scientific, USA) alone with D-Glucose and yeast extract were used during fed—batch Fermentation. Antifoam C emulsion (Sigma-Aldrich, St. Louis, MO, USA) was diluted with sterile water prior to addition and used as required during fermentation.

### Strains, plasmids, and culture conditions

The information related to the bacterial strain and plasmid used in this study is listed in Table S1. *E.coli* BL21(DE3) (Lucigen, Middleton, WI, USA) was used as the host strain for recombinant protein expression. The pETDuet-1 plasmid (Novagen, Madison, WI, USA) was used as the expression vector. Cells were cultivated in LB medium for recombinant protein expression and CGP production. Carbenicillin was used as a selective marker for plasmid maintenance. The cyanophycin synthetase (*cphA*) gene from *Synechocystis* sp. PCC 6803 (UniProt accession: P73833) was cloned into the pETDuet-1 expression vector (Novagen, Madison, WI, USA) for heterologous expression in *E.coli* BL21(DE3). As shown in Fig. S1, the recombinant construct contained a T7 promoter, lac operator, ribosome binding site (RBS), and ampicillin resistance marker for recombinant protein expression and plasmid selection. The codon-optimized *cphA* gene sequence used in this study is provided in Figure S2. The *cphA* gene was inserted into the multiple cloning site (MCS) of the pETDuet-1 vector and transformed into competent *E. coli* BL21 (DE3) cells for recombinant protein production and CGP biosynthesis studies. For Western blot analysis, a second recombinant construct, pLQ171_pETDuet-1-*cphA*-EGFP (8,802 bp; approximately 8.8 kb), encoding the CphA-EGFP fusion protein, was used. The expected molecular weight of the CphA-EGFP fusion protein was approximately 124.2 kDa. The plasmid map of the *cphA*-EGFP fusion construct is provided in Figure S3, and details of the plasmid are listed in Table S1. The GFP tag enabled detection of the fusion protein using an anti-GFP antibody during Western blot analysis.

Recombinant *E. coli* strain was cultivated in LB medium consisting of Yeast Extract (5 g L^−1^), NaCl (10 g L^−1^) and tryptone (10 g L^−1^) together with Carbenicillin (100 µg L^−1^). The cultivation was carried out in Shaker with an agitation speed of 200 rpm at 37 ℃. When the culture reached the mid logarithmic phase, 1% (v/v) pre-inoculated seed culture was added into the 250 mL Erlenmeyer flask consisting of LB medium with the working volume of 50 mL. The fermenter consisted of optimized media with following constituent: yeast extract (5 g L^−1^); glucose (20 g L^−1^); KH_2_PO_4_ (13.3 g L^−1^); citric acid (1.7 g L^−1^); (NH_4_)_2_HPO_4_ (4 g L^−1^); MgSO_4_·7H_2_O (1.2 g L^−1^) along with 5 mL L^−1^ of trace elements: Na_2_B_4_O_7_·10H_2_O (0.02 g L^−1^); ZnSO_4_·7H_2_O (2.25 g L^−1^); CuSO_4_·5H_2_O (1 g L^−1^); FeSO4·7H2O (10 g L^−1^); MnSO_4_·4H_2_O (0.5 g L^−1^); (NH4)6Mo7O24·4H2O (0.1 g L^−1^); CaCl2·2H2O (2 g L^−1^) and Carbenicillin was supplemented at a concentration of 100 µg mL⁻1 during batch fermentation processes for plasmid maintenance.

### Batch and fed-batch fermentation

Both batch and fed-batch fermentation were carried out in the bioreactor (4L, Eppendorf, USA) with the working volume of 1.5 L. The process parameters like Temperature, pH, agitation, aeration, and dissolved oxygen (DO) were controlled automatically (Eppendorf, USA). The pH of the fermenter was maintained at 7.0 ± 0.1 by automated addition of hydrochloric acid (4 N) and 28% (v/v) NH₄OH through peristaltic pumps connected to the bioreactor control system during the batch phase. During the fed-batch phase, pH was primarily maintained through controlled feeding of concentrated glucose and nutrient solution, thereby minimizing the requirement for continuous hydrochloric acid addition. Similarly, the temperature of the fermenter was controlled at 37 °C by the continuous addition of water to heating pad. The dissolved oxygen (DO) level was maintained above 30% throughout fermentation using a cascade control strategy. DO was regulated automatically by adjusting agitation speed and aeration rate within the operating ranges of 200–1100 rpm and 0.01–1.0 VVM, respectively. Antifoam was added manually when necessary. When the optical density (OD600) reached the mid-exponential growth phase, lactose (1% w/v) was added for protein induction along with supplementation of phosphate (1 mM) and ribose (10 g L⁻^1^) to support CGP biosynthesis and cellular metabolism.

Fed-batch fermentation was carried out using Glucose as the substrate in the feeding solution at various concentrations of 600, 400, and 200 g L⁻^1^ together with tryptone (60 g L⁻^1^) and yeast extract (60 g L⁻^1^) using a linear feeding strategy.

### Analytical methods

A spectrophotometer (SmartSpec Plus Spectrophotometer, USA) at a wavelength of 600 nm was used to monitor bacterial cell growth during shake-flask, batch, and fed-batch fermentation processes. OD600 measurements were performed using appropriately diluted samples to maintain absorbance values below 1.0 prior to spectrophotometric analysis. Culture samples were centrifuged (Eppendorf, USA) at 5000 rpm for 5 min at 4 °C to collect cell pellets for dry cell weight (DCW) determination. The collected pellets were washed with distilled water and subsequently freeze-dried under vacuum conditions prior to DCW quantification.

Glucose as the primary substrate and acetic acid as the major byproduct formed during fermentation were quantified using a high-performance liquid chromatography (HPLC) system equipped with an Aminex HPX-87H column (Bio-Rad, USA) and a refractive index detector. The column temperature was maintained at 35 °C, and separation was carried out using 5 mM sulfuric acid as the mobile phase at a flow rate of 0.5 mL min⁻^1^. Each detected compound was quantified by comparison with corresponding analytical standards.

### CGP recovery and precipitation

**Fig. 2:**
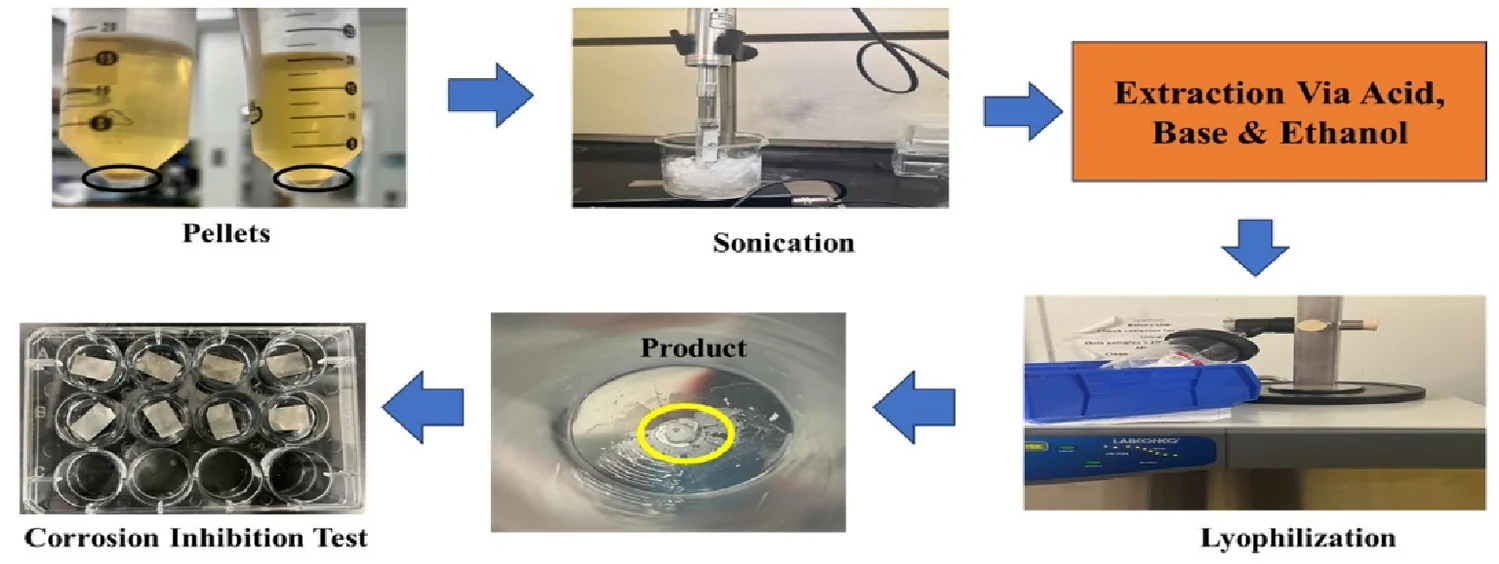
CGP recovery process and corrosion inhibition test

Since the sampling was performed every 3–4 h of the experiment. Whole recovery steps is shown in Fig. 2. Cell pellets were collected by centrifugation, resuspended in distilled water, and disrupted by sonication (QSonica, USA). Sonication was performed at 50% amplitude for 4 min using pulse conditions of 50 s ON and 10 s OFF under ice-cold conditions to minimize protein denaturation. The lysate pH was subsequently adjusted to 0.9 using 6 M HCl. The resulting mixture was centrifuged at 3500 **×** g for 10 min and the supernatant was collected, and pellet was discarded. The pH of supernatant was then adjusted to 7 via 2 M NaOH followed by centrifugation at 3500** ×** g for 10 min. This step resulted in the separation of Soluble CGP in supernatant and Insoluble CGP in pellets.

For soluble CGP, two volumes of ice-cold ethanol were added to supernatant for precipitation followed by centrifugation at 3500 × g for 10 min. The resulting pellet was resuspended in DI Water mixed with one volume of 95% ethanol and transferred to a pre-weighed 1 tube. After another round of centrifugation at 3500 × g for 10 min, the final pellet was collected for lyophilization and subsequent weighing. Similarly for insoluble CGP, the pellet containing insoluble CGP was processed by resuspending it in 0.1 M HCl and centrifuging at 3500 × g for 10 min. The pellet was discarded whereas the pH of supernatant was then neutralized with 2M NaOH followed by centrifugation at 3500 × g for 10 min. The resulting pellet, containing purified insoluble CGP, was also lyophilized and weighed. Because these recovered fractions were not subjected to complete purification prior to gravimetric measurement, the reported values represent crude CGP-containing fractions and may include residual cellular components, salts, moisture, and other co-extracted materials. Therefore, gravimetric values should not be interpreted as concentrations of pure intracellular CGP.

### CGP characterization

The chemical composition of freeze-dried CGP Powder was characterized using Fourier transform infrared (FTIR) spectroscopy (Shimadzu Corporation). The samples for FT-IR spectroscopic measurements were prepared outside the glovebox. The sample was mounted on the sample holder of a two-circle goniometer and enclosed within a radiation safety chamber. X-ray diffraction (XRD) analysis was performed using a 1 kW copper (Cu) X-ray tube operated at 40 kV and 25 mA. The measurement was conducted in the standard Bragg–Brentano para-focusing geometry, where X-rays emitted through a 1 mm divergence slit at the tube were directed toward the sample and subsequently focused onto a position-sensitive detector (Lynx-Eye, Bruker-AXS).MALDI mass spectrometry (Maxis 4G, Bruker Bioscience) analysis was performed to measure the mass spectrum in order to identify both soluble and insoluble CGP fragments. Both the soluble and insoluble fractions were detectable in positive and negative modes.

### Corrosion inhibition

Stainless steel sheets measuring 1.5 cm × 1 cm with a thickness of 0.002 inches were used for the experiment. Each sheet was weighed individually prior to treatment. The sheets were then immersed in various test solutions and incubated at 30 °C for different durations—specifically, 24, 48, and 72 h to assess the effects over time.

## Results and discussion

Certain polymer classes being developed via industrial fermentation could potentially replace existing petroleum-based polymers once production becomes cost-effective and/or measures are implemented to encourage the adoption of renewable resources. CGP is synthesized through a non-template-driven reaction catalyzed by CphA, which leads to a broad molecular weight distribution of the resulting biopolymer [16, 17]. Due to low polymer yield and slow growth rates, which result in low cell densities, various microorganisms like cyanobacteria are unsuitable for large-scale CGP production, and as a result, adequate quantities of CGP have not been available until now. However, *E. coli* is among the most widely used bacterial hosts for recombinant protein production, owing to its well-characterized metabolism and the availability of extensive genetic tools [18]. Advancements in genetic engineering have drawn significant interest toward developing high-yield CGP-producing *E. coli* strains using metabolic engineering and synthetic biology approaches [19].

### Recombinant CphA expression

**Fig. 3:**
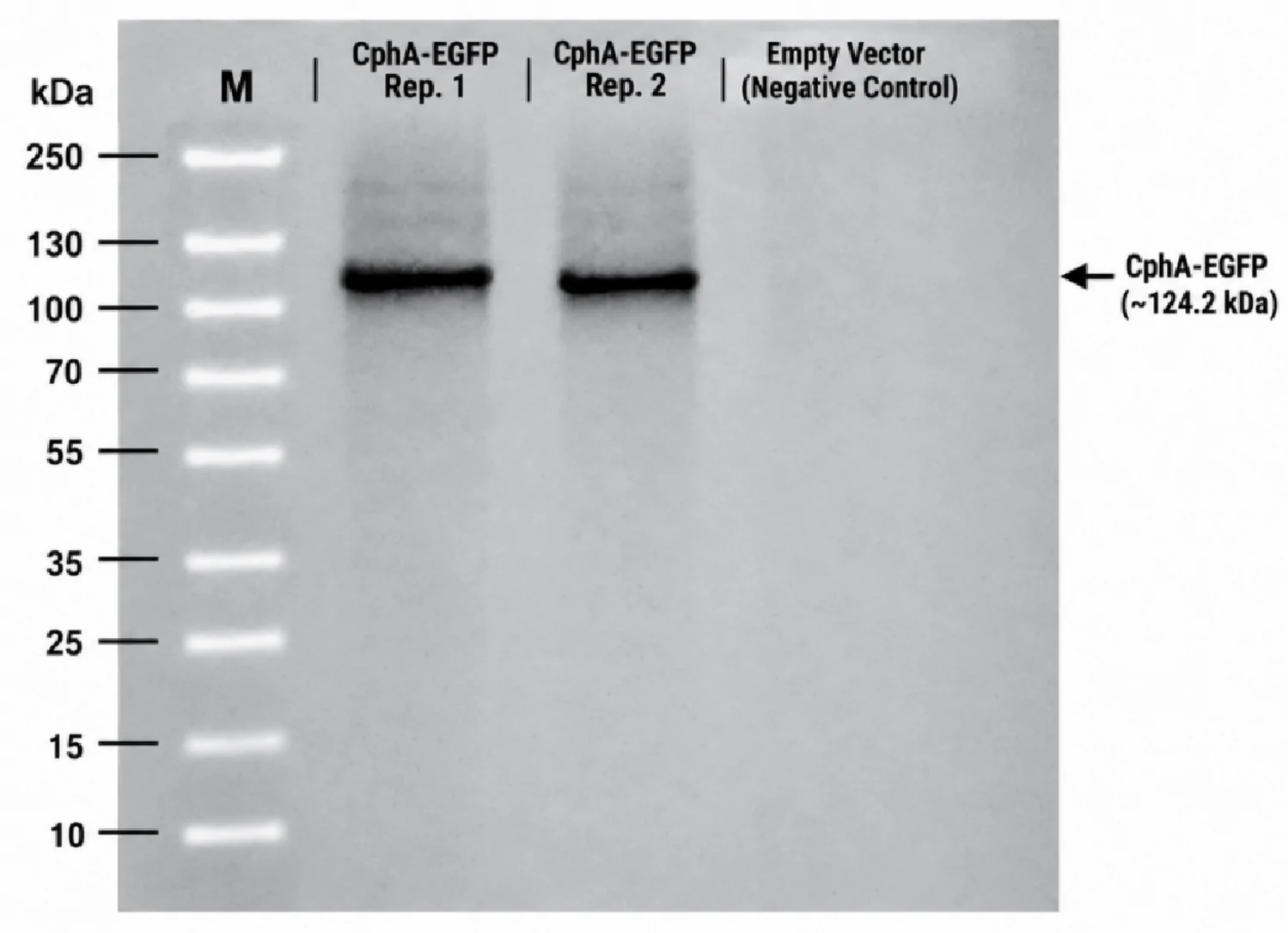
Western blot analysis of recombinant CphA-GFP fusion protein expression in recombinant *E. coli* BL21(DE3). Rep. 1 and Rep. 2 represent two replicate samples expressing the CphA-GFP fusion protein, and the empty vector was used as a negative control. The CphA-GFP fusion protein was detected using an anti-GFP antibody, with an observed band at approximately 120 kDa

Recombinant *E. coli* BL21 (DE3) expressing the CphA-EGFP fusion protein was analyzed by Western blot following lactose induction. The expected molecular weight of the CphA-EGFP fusion protein was approximately 124.2 kDa. Western blot analysis using an anti-GFP antibody showed a distinct immunoreactive band at approximately 120 kDa, consistent with the expected molecular weight of the CphA-EGFP fusion protein (Fig. 3), confirming expression of the recombinant fusion protein in the induced samples.

### Shake flask study for the production of CGP

**Fig. 4:**
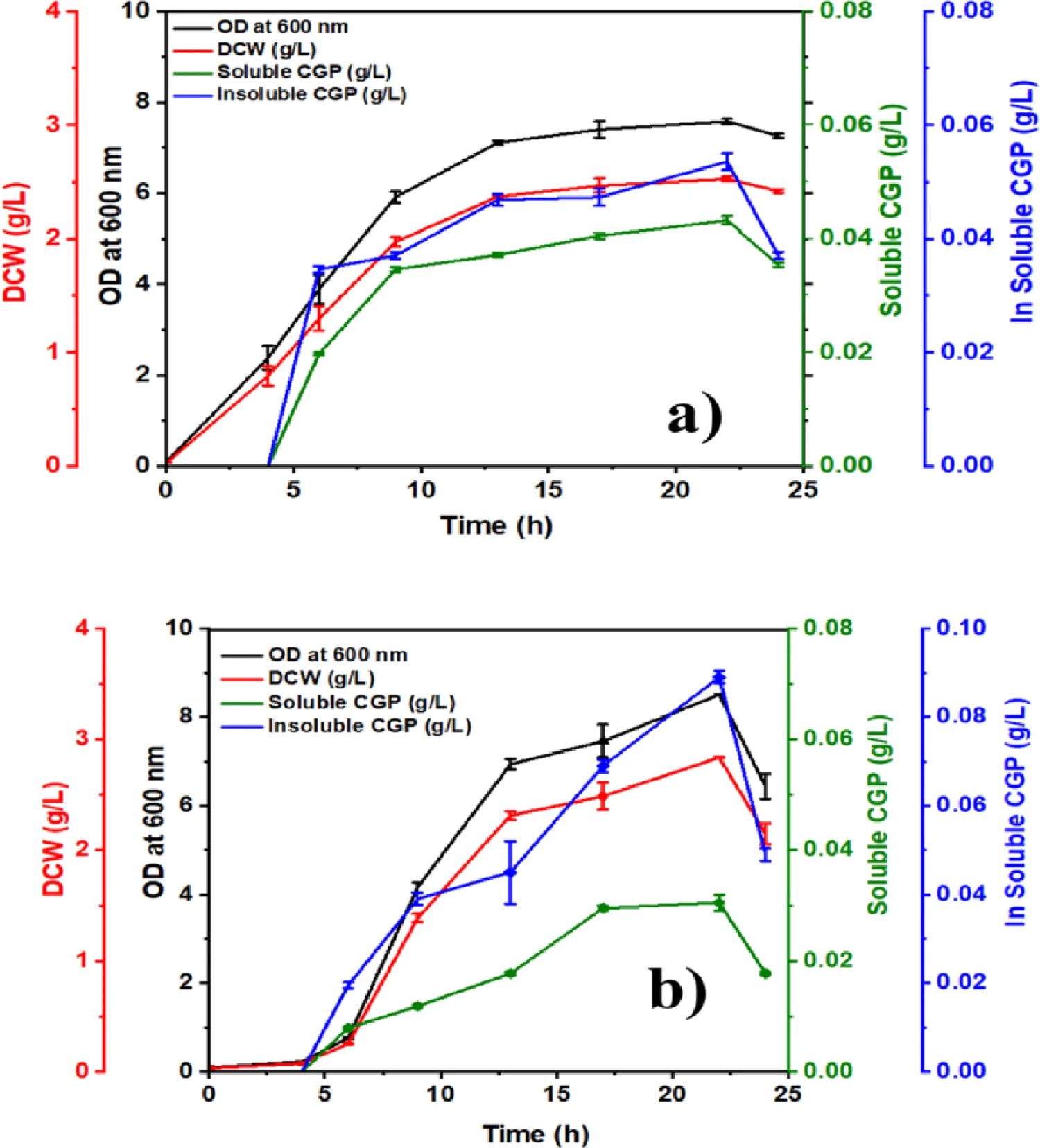
Time profiles of (**a**) bacterial growth, DCW, Soluble CGP production, Insoluble CGP production using IPTG as an Inducer, (**b**) bacterial growth, DCW, Soluble CGP production, Insoluble CGP production using lactose as an Inducer

Shake flasks are often employed for screening industrially promising microorganisms, media and process parameters optimization [20]. Their advantages include multiple benefits such as parallel processing, high flexibility, easy handling along with high experimental throughput. Considering the need to further scale up the cultivations, it was necessary to identify an alternative to IPTG as an inducer due to its high cost [13]. So, experiments were carried out using both IPTG (0.1 mM) and lactose 1% (w/v) an inducer. With IPTG as an inducer, the reaction lasted for 24 h with the highest optical density reaching 7 with the gravimetrically recovered crude soluble (0.042 g L^−1^) and insoluble (0.05 g L^−1^) CGP. However, with the lactose as an inducer with the same reaction time, the optical density slightly increased along with the gravimetrically recovered crude soluble (0.037 g L^−1^) and insoluble (0.09 g L^−1^) CGP respectively (Fig. 4a, b). These results indicate that lactose induction is more effective than IPTG and could contribute to reducing overall production costs at the industrial scale. A similar study was carried out by Maharjan et al. [21] for the production of biochemical compounds using the same inducing agents with metabolically recombinant *E. coli.* An increase in production and optical density was also observed when lactose was used as the inducer compared to IPTG. In addition, previous studies have also demonstrated the successful use of lactose as both an inducer and carbon source for CGP biosynthesis in recombinant *E. coli* systems. Kroll et al. [13] reported large-scale CGP production using recombinant *E. coli*, where lactose was continuously used as an effective inducer and carbon source during fermentation.

Shake flasks do not support real-time monitoring and rely on surface aeration, which is limited by diffusion constraints [22]. With the primary goal of optimizing process parameters through submerged cultivation using low-cost inducers, further study was conducted.

### Batch mode bioreactor setup and operation

**Fig. 5:**
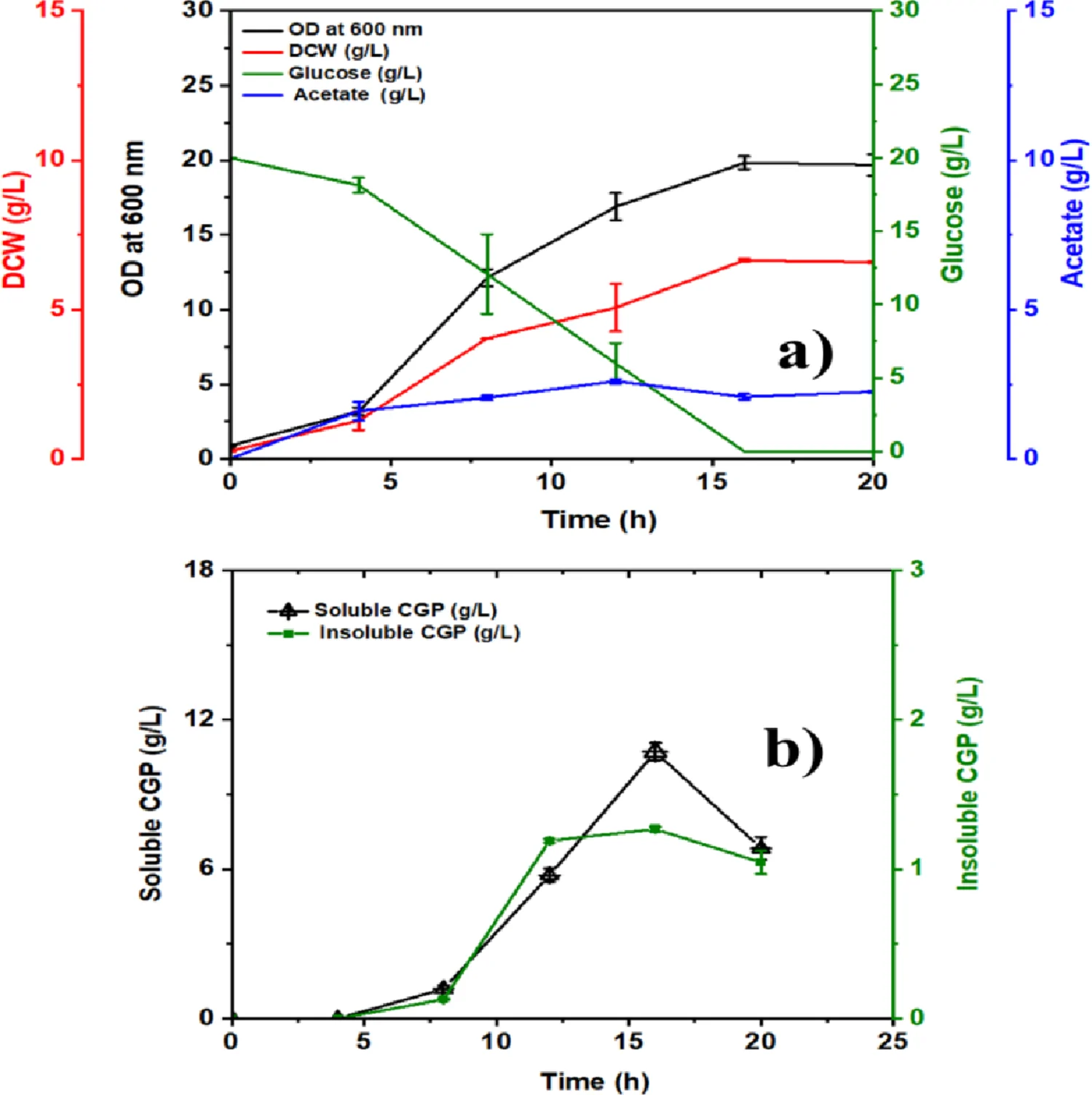
Time profiles of (**a**) bacterial growth, DCW, glucose consumptions, Acetate accumulation (**b**) soluble CGP production, Insoluble CGP production without phosphate and ribose supplementation. Time profiles of (**a**) bacterial growth, dry cell weight (DCW), glucose consumption, and acetate accumulation; and (**b**) soluble and insoluble crude CGP-containing fractions without phosphate and ribose supplementation

Batch experiments were conducted in two stages: the pre-induction phase and the post-induction phase. Initially, no notable cell growth was observed due to alterations in the specific growth rates during the pre-induction phase, which subsequently impacted growth after induction [23, 24]. Using the optimized shake flask conditions, batch fermentation was carried out with 20 g/L glucose as the sole carbon source. The maximum optical density of approximately 20 was achieved at 18 h. Glucose was fully consumed by around 16 h, along with acetate production of approximately 2.2 g/L. Product formation began after induction, resulting in gravimetrically recovered crude soluble and insoluble CGP-containing fractions of 10.71 and 1.268 g/L, respectively (Fig. 5a, b).In this case, pH of the fermenter was controlled at (7.00 ± 0.1) by the automated addition of HCl (4 N) and NaOH (4 N) through the peristaltic pumps connected to the control system.

Micronutrients play a crucial role in microbial metabolism, contributing to enhanced biomass yield and overall productivity [25]. Their supplementation has been shown to improve both biomass concentration and bio product formation [26, 27]. When ammonium hydroxide was used both as a pH control agent and a nitrogen source, maximum biomass concentration and efficient substrate utilization were observed [28]. Moreover, phosphates have diverse effects on enzyme activity and structure, acting as activators of both bacterial and mammalian synthetases. However, high phosphate concentrations can be inhibitory, as they compete with ribose−5-phosphate binding and promote inhibition through ADP inhibitors [28]. In addition to glucose, ribose supplementation was investigated as a metabolic supplement to support biomass accumulation and CGP biosynthesis. Ribose can participate in central carbon metabolism through the pentose phosphate pathway, potentially contributing to precursor generation and cellular energy metabolism associated with recombinant protein production [40]. Although ribose is relatively more expensive than glucose, only a limited concentration (10 g L⁻^1^) was supplemented during cultivation. Under the tested conditions, ribose supplementation was associated with improved biomass accumulation and enhanced CGP production performance. Based on these hypotheses and the optimized parameters reported by Maharjan et al. [21], further batch mode experiments were conducted using phosphate and ribose supplementation to evaluate their effects on biomass accumulation and CGP production.

**Fig. 6:**
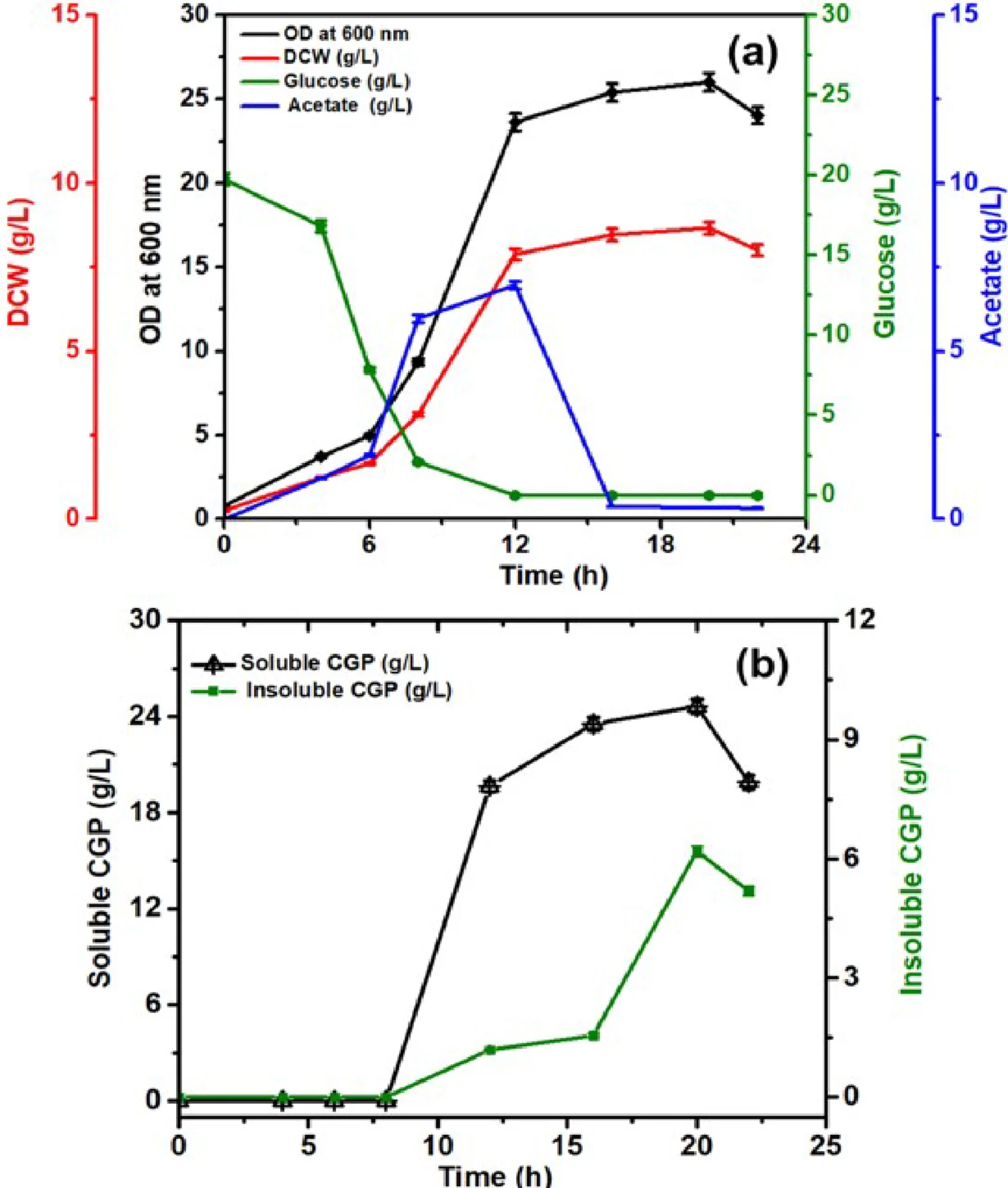
Time profiles of (**a**) bacterial growth, DCW, glucose consumptions, Acetate accumulation (**b**) soluble CGP production, Insoluble CGP production with phosphate and ribose supplementation

The adoption of these supplementations led to a two-fold increase in the maximum optical density. Glucose consumption was completed within 8–10 h of the experiment, accompanied by acetate production of approximately 7 g/L. Following glucose depletion, acetate was also utilized within the system. Furthermore, combined addition of phosphate and ribose resulted in a 2.24-fold increase in the gravimetrically recovered crude soluble CGP-containing fraction and a 4.88-fold increase in the gravimetrically recovered crude insoluble CGP-containing fraction (Fig. 6a, b). With the primary objective of enhancing cell density and production through efficient substrate utilization, fed-batch fermentation was employed, as discussed below.

### Fed-batch fermentation for high-cell-density cultivation and CGP production

Various feeding strategies such as pH–stat [29], exponential feeding, and linear feeding [30] have been applied to achieve high cell density and enhanced bioproduct formation in recombinant *E.coli* systems. In the present study, a linear feeding strategy was selected because of its operational simplicity and practicality compared with more complex feeding approaches such as continuous or DO-stat feeding. This strategy enabled gradual substrate addition during fed-batch cultivation without the requirement for external pure oxygen sparging or highly precise automated feeding control systems. In addition, previous studies have reported that gradual substrate feeding can help limit excessive glucose accumulation and uncontrolled rapid biomass growth, thereby reducing oxygen demand during cultivation [23]. Since glucose serves as the primary carbon source for CGP biosynthesis, the effect of feed-glucose concentration on biomass accumulation and product formation was investigated. The glucose concentration of the feeding solution (600 g L⁻^1^) was selected based on previously reported high-cell-density fed-batch fermentation studies using recombinant *E. coli* for bioproduct biosynthesis [29, 30]. Subsequently, lower feed-glucose concentrations of 400 and 200 g/L were evaluated to reduce residual glucose accumulation and improve substrate utilization during fermentation. In addition, yeast extract and tryptone were supplemented in the feeding solution to enrich the nutrient composition. Previous studies have reported that supplementation with yeast extract and tryptone can enhance biomass accumulation and CGP production in recombinant *E. coli* cultures [31]. The linear feeding strategy applied in this study should be considered as a preliminary feed-concentration screening approach rather than a fully optimized fed-batch control strategy. Accordingly, the study aimed to evaluate glucose utilization behavior, biomass accumulation, and CGP production performance under different feeding-solution glucose concentrations in the recombinant *E. coli* system.

Fed-batch fermentation was performed in three distinct stages. The first stage involved monitoring the duration required for complete glucose depletion in the reactor. Once the optical density (OD) reached approximately 8–10, lactose induction was carried out, followed by the addition of macronutrients to boost production and cell density. The final stage involved applying linear fed-batch fermentation.

**Fig. 7:**
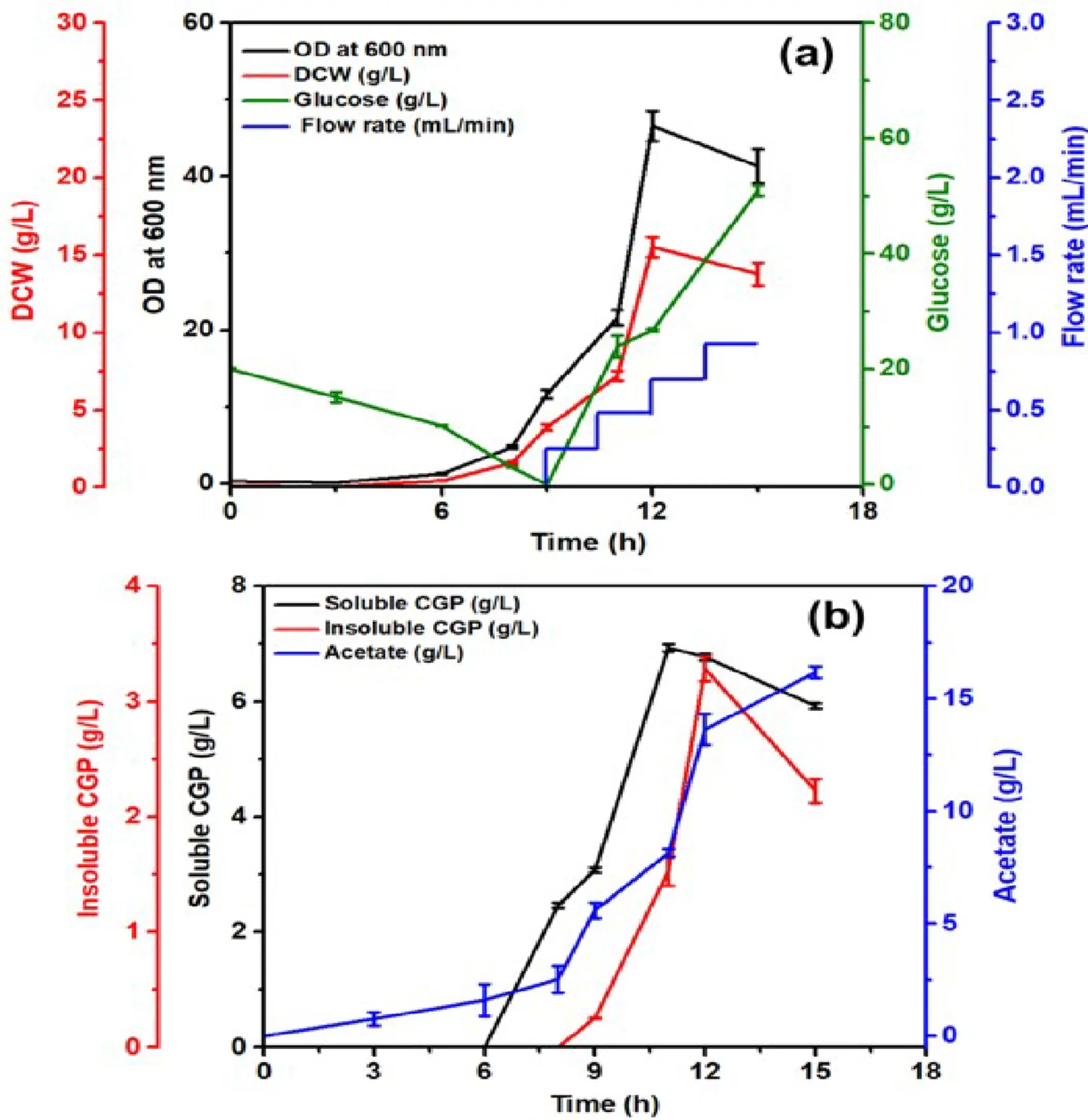
Time profile of (**a**) bacterial growth, DCW, glucose consumptions, Feeding rate (**b**) soluble CGP production, Insoluble CGP production, Acetate Accumulation (600 g/L Glucose)

**Fig. 8:**
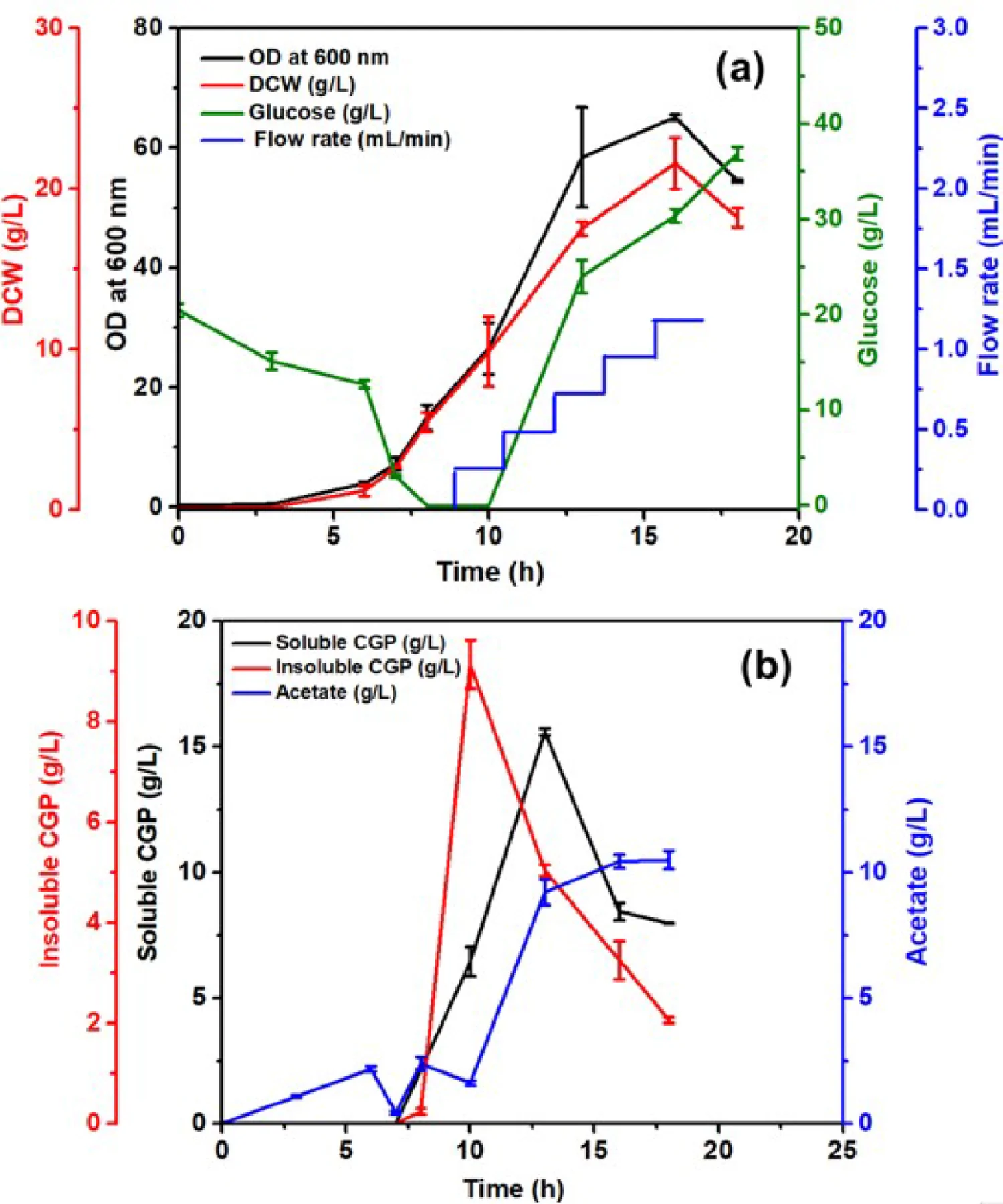
Time profile of (**a**) bacterial growth, DCW, Glucose consumptions, Feeding rate (**b**) Soluble CGP production, Insoluble CGP production, Acetate Accumulation (400 g/L Glucose)

**Fig. 9:**
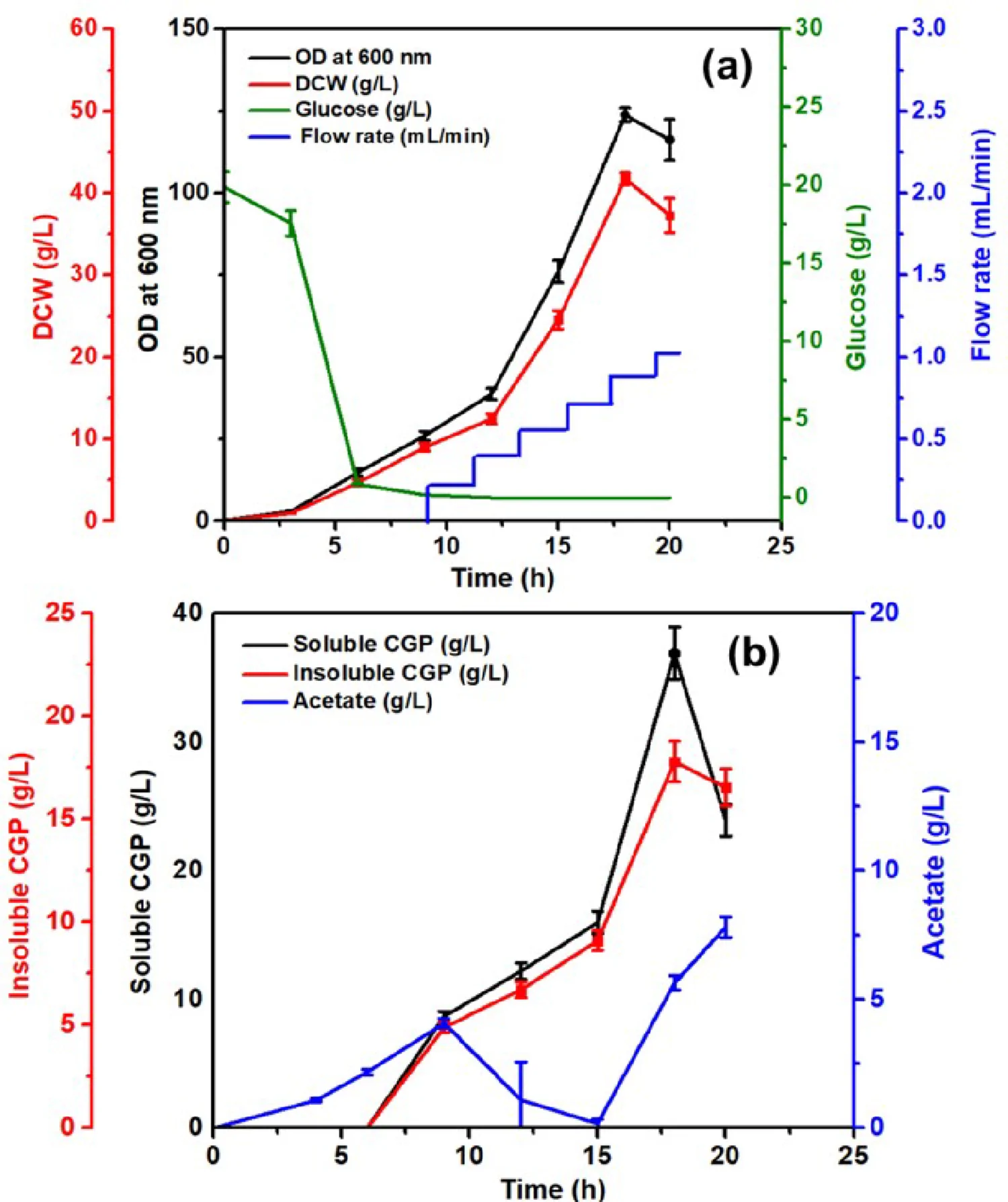
Time profile of (**a**) bacterial growth, DCW, Glucose consumptions, Feeding rate (**b**) Soluble CGP production, Insoluble CGP production, Acetate Accumulation (200 g/L Glucose)

The impact of different glucose concentrations in the feeding solutions (600, 400, and 200 g L⁻^1^) on CGP production was investigated, as illustrated in Figs. 7, 8, and 9. Using a feeding solution containing 600 g L⁻^1^ glucose, the maximum optical density of approximately 47 was achieved around 12 h of cultivation. Feeding was initiated after complete depletion of the initial glucose present in the reactor medium, which occurred at approximately 8 h of cultivation. However, residual glucose accumulation and increased acetate formation were observed during fed-batch cultivation under this condition. The gravimetrically recovered crude soluble and insoluble CGP-containing fractions reached 6.935 and 3.28 g L⁻^1^, respectively (Fig. 7a, b).

In the second run, the glucose concentration in feeding solution was reduced to 400 g/L. With this reduction, glucose was initially consumed; however, after a certain period, it began to accumulate again, accompanied by acetate accumulation. Despite this, the optical density increased by 1.40-fold, while the gravimetrically recovered crude soluble and insoluble CGP-containing fractions increased by 2.25-fold and 1.54-fold, respectively (Fig. 8a, b).

In the third run, the glucose concentration in feeding solution was further reduced to 200 g/L. Under these conditions, all the glucose fed into the reactor was completely consumed, resulting in the highest cell density of 126. This was accompanied by maximum gravimetrically recovered crude soluble and insoluble CGP-containing fractions of 35.4 and 17.8 g/L, respectively. However, cell growth was inhibited due to accumulation of acetate within the system (Fig. 9a, b).

Acetate accumulation was observed during fed-batch cultivation under all glucose feeding conditions, with higher glucose feed concentrations resulting in greater acetate formation. In recombinant *E.coli* systems, acetate accumulation commonly occurs under high substrate uptake and overflow metabolism conditions associated with excess acetyl-CoA formation [23]. Excessive accumulation may negatively affect biomass accumulation and cellular metabolic activity, thereby reducing intracellular CGP production performance. In the present study, the 200 g/L glucose feeding condition resulted in lower acetate accumulation, improved biomass formation, and increased recovery of crude CGP-containing fractions compared with higher glucose feeding concentrations.

To further evaluate the influence of feed-glucose concentration on fermentation performance, apparent kinetic and yield parameters including specific growth rate (µ), specific substrate uptake rate ($${\mathrm{q}}_{\mathrm{s}}$$), specific product formation rate ($${\mathrm{q}}_{\mathrm{p}}$$), biomass yield on substrate ($${Y}_{\raisebox{1ex}{$X$}\!\left/ \!\raisebox{-1ex}{$S$}\right.}$$), and product yield on substrate ($${Y}_{\raisebox{1ex}{$\mathrm{P}$}\!\left/ \!\raisebox{-1ex}{$\mathrm{S}$}\right.}$$), were calculated for each feeding condition (Table 1). Among the tested conditions, the 200 g/L feed concentration exhibited the highest specific growth rate and yield coefficients, indicating improved substrate utilization efficiency and increased recovery of crude CGP-containing fractions compared with higher glucose feed concentrations.

**Table 1:** Effect of feed-glucose concentration on kinetic and yield parameters during fed-batch fermentation

Feed glucose	µ (h⁻^1^)	${\mathrm{q}}_{\mathrm{s}}$ g glucose g⁻^1^ DCW h⁻^1^	${\mathrm{q}}_{\mathrm{p}}$ g CGP g⁻^1^ DCW h⁻^1^	${\mathrm{Y}}_{\mathrm{x}/\mathrm{s}}$ g DCW g glucose⁻^1^	${Y}_{\mathrm{p}/\mathrm{s}}$ g CGP g glucose⁻^1^	CGP (g/L)
200 g/L	0.24	0.35	0.099	0.326	0.281	53.2
400 g/L	0.19	0.91	0.067	0.115	0.074	20.65
600 g/L	0.11	1.69	0.106	0.135	0.063	10.25

### Recovery, corrosion inhibition and characterization of CGP

**Fig. 10:**
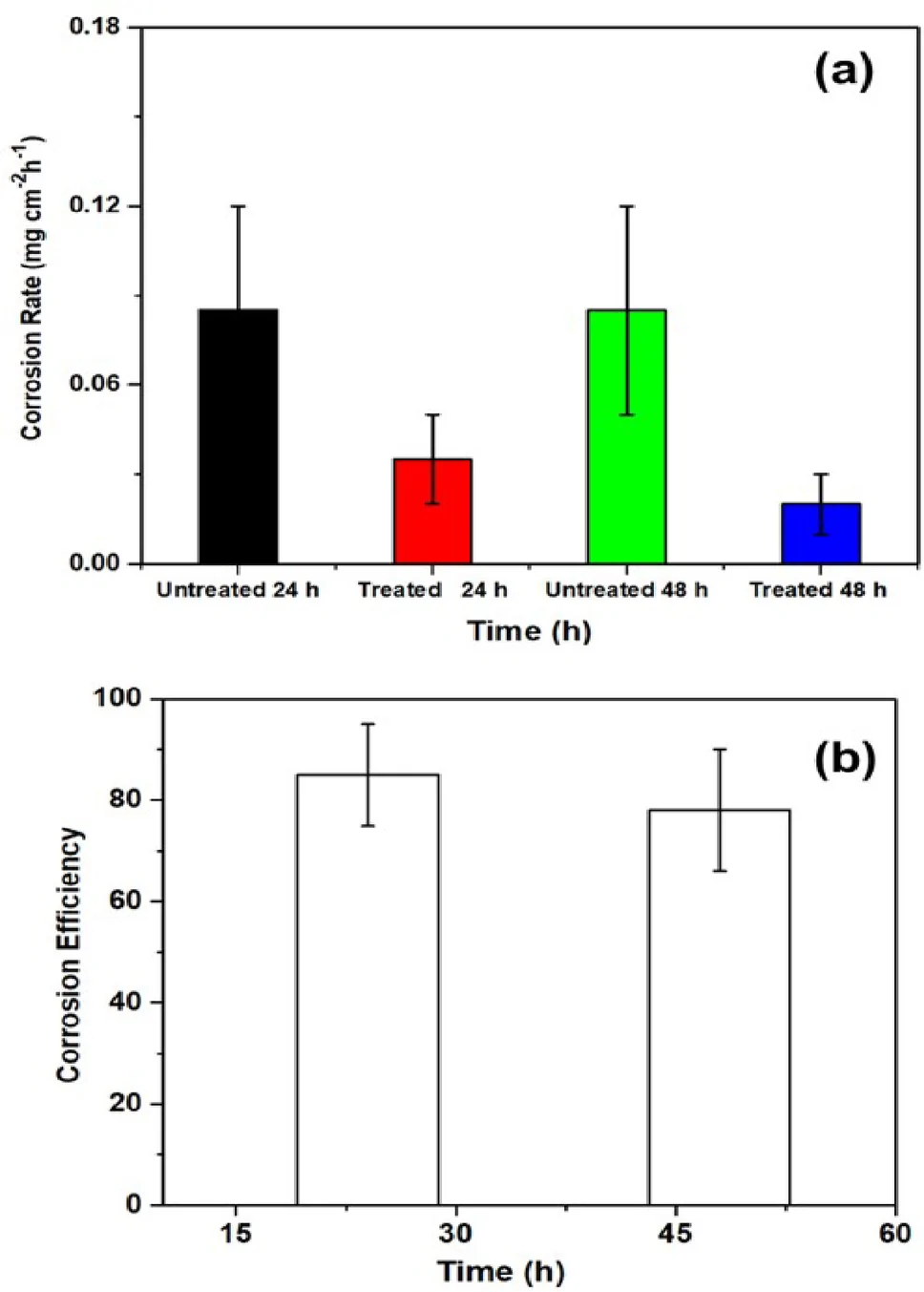
Time profile of (**a**) Corrosion Rate (**b**) Corrosion Inhibition Efficiency

**Fig. 11:**
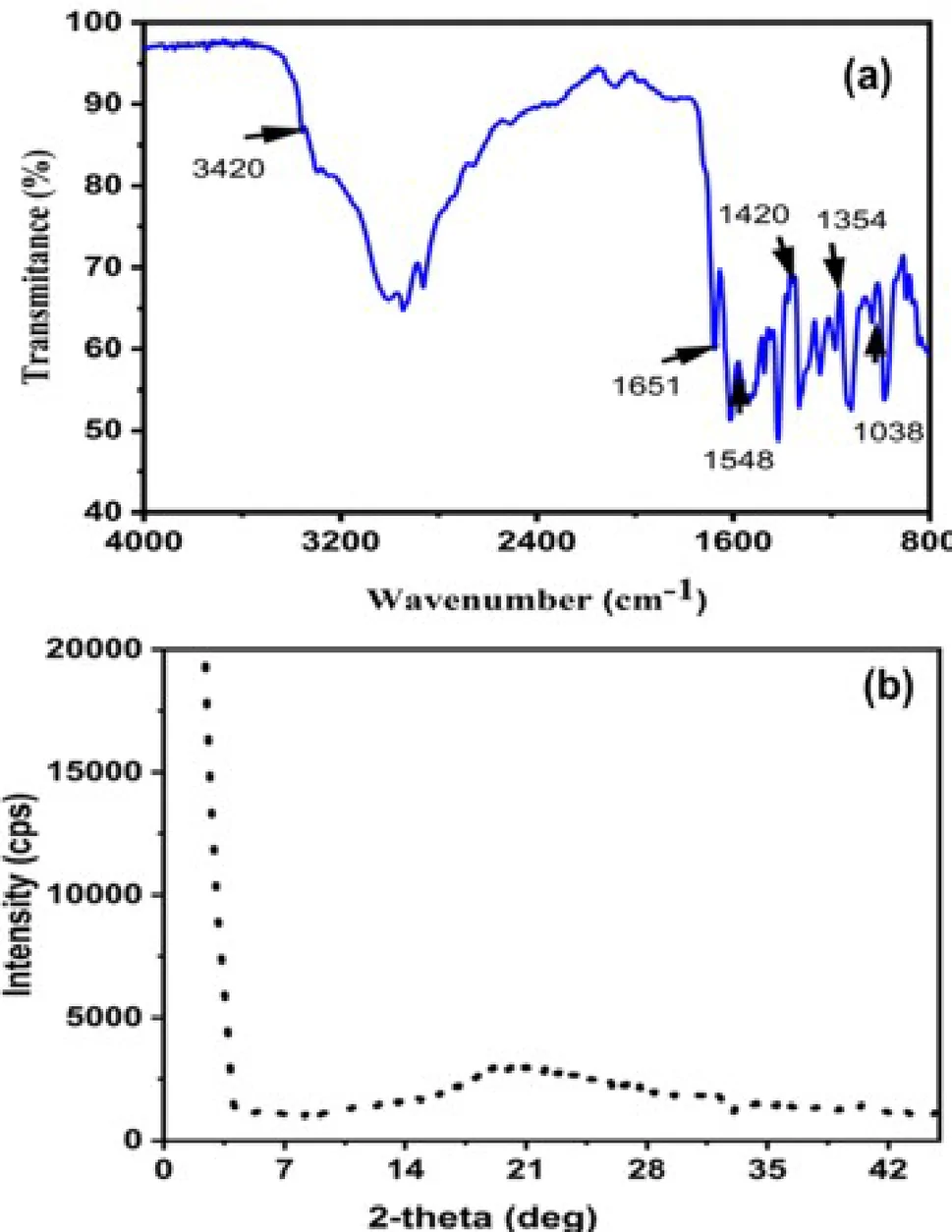
CGP qualitative analysis (**a**) FTIR analysis (**b**) XRD analysis

The CGP recovery procedure was described in the Methods section. Samples were collected at various time intervals, weighed using an analytical balance, and expressed in grams per liter (g/L). A similar approach has been reported in the literature [32, 33]. Furthermore, corrosion inhibition tests were performed as described in the Materials and Methods section, resulting in a corrosion inhibition efficiency of 85% and a corrosion rate of 0.082 mg·cm⁻^2^·h⁻^1^ (Fig. 10a, b). Figure 11a illustrates the FTIR peaks of the extracted CGP, with notable absorption bands observed around 3420 cm⁻^1^ (attributed to O–H and N–H groups), 1651 cm⁻^1^ (corresponding to C=N in guanidyl and C=O in amide I), 1548 cm⁻^1^ (related to N–H bending), 1420 cm⁻^1^ (associated with C–N stretching), 1354 cm⁻^1^ (indicative of COO⁻ groups), and 1038 cm⁻^1^. This similarity indicates that the CGP in our sample exhibits comparable characteristics to those reported in references [34, 35]. XRD analysis was also performed for the crystallinity of the powder products. According to [36, 37], CGP exhibits an amorphous solid structure, similar to the behavior observed in polymethacrylate and polyvinyl sulfobetaines. Consistent with this, our results (Fig. 11b) show no evidence of crystalline structures, indicating that the charged side chains of CGP do not form crystalline arrangements in the solid state. MALDI-MS analysis (Fig. S4) was performed to further characterize the recovered CGP fractions obtained from recombinant *E. coli* cultures. Distinct mass distribution patterns were observed between the negative control (empty vector) and CGP-producing strains in both positive and negative ionization modes. The soluble CGP fraction exhibited molecular weight distributions primarily within the range of approximately 10–25 kDa, whereas the insoluble CGP fraction showed relatively higher molecular weight distributions in the range of approximately 20–25 kDa. Several lower-molecular-weight fragment peaks were also observed, which may be associated with partial fragmentation of CGP during sample preparation or ionization analysis.

Compared with the negative control samples, CGP-producing strains demonstrated significantly stronger characteristic mass signal distributions, supporting successful biosynthesis and recovery of CGP in both soluble and insoluble fractions.

Productivity is strongly influenced by cell density during microbial fermentation. High-cell-density cultivation in bioreactors can significantly enhance intracellular CGP accumulation, facilitate downstream recovery, and potentially reduce production costs at the industrial scale [38]. In the present study, fed-batch fermentation resulted in maximum gravimetrically recovered crude soluble and insoluble CGP-containing fractions of 35.4 and 17.8 g/L, respectively, together with a corrosion inhibition efficiency of 85%.

As summarized in Table 2, the gravimetrically recovered crude CGP-containing fractions obtained in this study were higher than those reported in several previous recombinant *E. coli* studies using cost-effective induction strategies. However, direct comparison should be made with caution because differences in CGP recovery, purification, and quantification methods may influence the reported values. Furthermore, industrial-scale implementation and large-scale process validation have not yet been demonstrated. Therefore, the present study provides a preliminary framework for future research focused on scalable and cost-effective CGP production and the development of biologically derived corrosion-resistant materials using fed-batch *E. coli* fermentation systems.

**Table 2:** Comparison of reported CGP production systems under different strains and cultivation conditions

Strain	System	Mode	Soluble CGP (g/L)	Insoluble CGP (g/L)	Total CGP (g/L)	References
*E. coli* DH1	Bioreactor	Batch	–	–	1.44	[32]
Cyanobacteria strain BW86	Photobioreactor	Batch	–	–	1	[39]
*E. coli* BL21(DE3)	Shake flask	Flask	–	–	1.7	[35]
*E. coli* BL21 (DE3)	Bioreactor	Fed-batch	35.4	17.8	53.2	This study

## Conclusions

CGP production was successfully demonstrated in a 4-L fed-batch bioreactor with a 1.5-L working volume using recombinant *E. coli*, together with a cost-effective lactose-based induction strategy and selected nutrient supplementation. Under fed-batch cultivation conditions, maximum gravimetrically recovered crude soluble and insoluble CGP-containing fractions of 35.4 and 17.8 g/L, respectively, were obtained.

An adapted downstream recovery process based on acid/base extraction and ethanol precipitation enabled successful recovery of CGP-related material for further characterization. Product characterization was supported by FTIR, XRD, and MALDI-MS. In addition, preliminary corrosion inhibition experiments under acidic conditions demonstrated reduced corrosion behavior in the presence of the recovered material compared with untreated controls.

Overall, this study demonstrates the potential of recombinant *E. coli* for high-cell-density CGP production using a cost-effective induction strategy and provides a foundation for future optimization of upstream cultivation, downstream purification, and corrosion-related applications on a larger scale.

## Supplementary Information

Below is the link to the electronic supplementary material.

Supplementary Material 1

## Data Availability

Data will be available on request.
