# Supplementary material for: Biosynthesis of cyanophycin by high-cell-density cultivation of recombinant Escherichia coli for corrosion-resistant biomaterial applications
Source: Bioprocess Biosyst Eng. 2026 Aug 21;49(10):2777–91. doi: 10.1007/s00449-026-03412-9 (PMC13582230; doi:10.1007/s00449-026-03412-9)
Supplement: Supplementary file 1 [file 449_2026_3412_MOESM1_ESM.docx]

**Supplementary data**

**Biosynthesis of Cyanophycin by High-Cell-Density Cultivation of Recombinant *Escherichia coli* for Corrosion-Resistant Biomaterial Applications**

Saroj Raj Kafle, Anirudh Mukunth, Arum Han, Arul Jayaraman, Aristos Aristidou*

Artie McFerrin Department of Chemical Engineering, Texas A&M University, TX, 77840

**^*^Corresponding author:**

Email: [**aristidou@tamu.edu**](mailto:aristidou@tamu.edu)

**Figure S1** Schematic diagram representing the recombinant pETDuet-1-*cphA* expression plasmid constructed for cyanophycin production in *Escherichia coli* BL21. The codon-optimized *cphA* gene from *Synechocystis* sp. PCC 6803 was cloned into the multiple cloning site (MCS) of the pETDuet-1 vector under the control of the T7 promoter and lac operator system. The plasmid also contains a ribosome binding site (RBS), ampicillin resistance marker (AmpR), and f1 origin for plasmid replication and selection. The plasmid map was generated using SnapGene software

**Annotation:** 5′ flanking region (gray highlight); **ATG start codon**; cphA coding sequence (unhighlighted); **TAA stop codon**; 3′ flanking region (gray highlight).

GCCATCACCATCATCACCACAGCCAGGATCCGAATTCG**ATG**AAGATTTTAAAGACGTTGACTCTTCGCGGACCCAACTACTGGTCAATCCGTCGTAAAAAGTTAATCGTTATGCGTTTGGATCTGGAAGACCTTGCCGAACGTCCGAGTAATTCGATTCCCGGGTTTTATGAGGGATTGATTCGTGTCTTACCCTCCCTGGTTGAGCATTTCTGCAGCCCCGGACACCGTGGGGGATTTCTTGCACGCGTCCGTGAGGGTACGTATATGGGACACATCGTGGAGCATGTTGCATTGGAATTACAAGAACTGGTAGGGATGACGGCCGGCTTCGGCCGCACCCGTGAGACTTCCACTCCGGGAATCTATAATGTCGTGTATGAGTATGTTGATGAACAGGCAGGCCGTTATGCGGGGCGTGCCGCTGTACGTTTATGCCGTAGTCTGGTGGACACGGGGGATTATTCTTTGACCGAGTTGGAAAAAGATCTGGAGGATCTTCGCGATCTTGGCGCAAACTCTGCTTTGGGACCTAGTACCGAAACAATCGTGACTGAGGCAGACGCGCGCAAAATTCCATGGATGCTGTTGTCCGCGCGCGCAATGGTTCAGCTGGGGTATGGCGTCCACCAGCAGCGTATTCAGGCCACTCTGTCAAGCCACTCCGGTATCCTTGGGGTGGAGCTGGCATGTGACAAAGAAGGAACCAAAACGATTTTGCAGGATGCCGGTATTCCCGTCCCGCGCGGCACGACGATTCAGTACTTCGACGACTTAGAAGAGGCTATCAATGACGTTGGCGGTTACCCGGTAGTGATTAAGCCACTGGATGGGAATCATGGCCGTGGAATCACGATTAACGTGCGTCACTGGGAGGAAGCTATCGCAGCATACGATCTTGCAGCCGAGGAATCGAAATCGCGTTCAATTATTGTAGAGCGCTATTATGAAGGGAGTGATCACCGCGTCTTGGTAGTGAACGGTAAGCTGGTCGCTGTCGCTGAACGTATTCCTGCACACGTGACTGGAGATGGGACGTCCACTATCACTGAATTGATTGACAAGACTAATCAAGATCCGAACCGTGGGGATGGGCATGCCAACATCTTGACAAAGATTGTCGTGAACAAAACCGCTATCGATGTCATGGAACGTCAAGGGTACAATCTTGACTCGGTGTTGCCAAAAGACGAAGTCGTTTACTTGCGTGCGACAGCAAATTTATCTACTGGCGGGATTGCAATTGACCGCACTGATGACATCCACCCTGAAAATATCTGGTTGATGGAGCGTGTAGCAAAAGTCATTGGGTTGGATATTGCCGGTATCGATGTCGTAACCTCAGATATCTCGAAACCGTTGCGTGAAACGAATGGCGTGATCGTAGAGGTAAACGCCGCGCCAGGCTTCCGTATGCATGTGGCACCTTCCCAGGGTCTTCCTCGCAACGTAGCAGCCCCCGTATTGGACATGTTATTTCCGTCAGGTACACCCTCCCGCATCCCAATTCTTGCGGTAACTGGGACCAATGGCAAGACCACGACAACTCGTTTGTTAGCTCACATTTACCGTCAGACGGGGAAAACTGTGGGATACACTTCCACCGACGCTATTTACATCAATGAGTACTGCGTTGAAAAAGGCGATAATACTGGCCCACAGTCTGCCGCAGTAATCCTTCGTGATCCGACCGTAGAAGTTGCGGTGCTTGAGACTGCTCGTGGCGGAATCCTTCGCGCGGGTTTGGCTTTCGATACGTGCGATGTGGGGGTGGTGTTGAACGTAGCCGCTGACCATCTGGGGCTTGGCGATATTGATACGATTGAACAGATGGCTAAGGTCAAATCGGTCATTGCAGAGGTAGTAGATCCGTCTGGTTACGCTGTACTTAATGCGGACGATCCGTTAGTTGCCGCAATGGCTGATAAAGTGAAAGCGAAGGTGGCATATTTCAGTATGAACCCGGACAATCCGGTCATCCAGAATCATATTCGCCGTAACGGTATTGCTGCTGTGTACGAGTCTGGATACGTGAGCATCTTGGAGGGCTCGTGGACGCTGCGCGTGGAAGAGGCCACTTTGATCCCGATGACCATGGGTGGGATGGCTCCATTCATGATCGCCAATGCCTTAGCTGCTTGCTTAGCTGCCTTCGTGAATGGATTAGATGTTGAAGTTATCCGCCAAGGAGTCCGTACGTTCACTACGTCCGCAGAGCAAACTCCCGGTCGTATGAATTTATTTAACCTGGGACGCTACCACGCTTTGGTGGATTATGCCCACAATCCAGCGGGATACCGCGCCGTCGGGGACTTCGTTAAGAACTGGCACGGCCAACGTTTCGGCGTCGTCGGGGGACCAGGAGACCGCCGCGATTCAGATTTGATCGAATTAGGTCAAATTGCGGCTCAGGTGTTCGACCGCATTATTGTCAAAGAGGACGACGACAAACGCGGCCGTTCGGGGGGAGAAACAGCAGACCTGATCGTCAAGGGTATCCTTCAGGAGAACCCGGGAGCGGCTTATGAAGTCATTCTTGACGAGACTGTAGCTCTGAACAAGGCATTAGATCAGGTCGAAGAAAAGGGTTTGGTTGTGGTTTTCCCAGAGTCTGTATCAAAAGCGATCGAACTTATTAAGGCTCGTAAACCGATCGGA**TAA**AGCTCGGCGCGCCTGCAGGTCGACAAGCTTGCGGCCGCATAATGCTTAAGTCGAACAGAAAGTAATCGTA

**Figure S2** Codon-optimized DNA sequence of the cphA gene encoding cyanophycin synthetase from Synechocystis sp. PCC 6803. The cphA coding-sequence boundaries are indicated by the annotated ATG start codon and TAA stop codon, while the 5′ and 3′ flanking sequences used for cloning are indicated separately.

**Figure S3** Plasmid map of the pLQ171_pETDuet-1-*cphA*-EGFP construct (8,802 bp) used for expression of the CphA-EGFP fusion protein in *E. coli* BL21 We thank the reviewer for pointing out this inconsistency. We have corrected the strain designation from *E. coli* BL21 to *E. coli* BL21(DE3) in Line 79, Table S1, and throughout the revised manuscript and Supplementary Information. The construct contains the *cphA* gene fused to superfolder GFP through a linker sequence.

**Figure S4** MALDI-MS analysis of soluble and insoluble CGP fractions obtained from recombinant *E. coli*. (a) Positive ion mode analysis of soluble CGP fraction compared with soluble fraction from empty vector control. (b) Negative ion mode analysis of soluble CGP fraction compared with negative control. (c) Positive ion mode analysis of insoluble CGP fraction compared with insoluble fraction from negative control. (d) Negative ion mode analysis of insoluble CGP fraction compared with negative control. The detected mass distribution patterns indicated soluble CGP fragments predominantly within the range of approximately 10–25 kDa, while insoluble CGP fractions showed higher molecular weight distributions around 20–25 kDa.

**Table S1** Detailed description of strains, genes, and plasmids used in this study.

| S.N. | Strain, genes, and plasmids | Description |
| --- | --- | --- |
| 1 | Host strain (*Escherichia coli* BL21 DE3) | Used as the recombinant protein expression host for cyanophycin synthetase (*cphA*) expression and CGP production |
| 2 | Cyanophycin synthetase (*cphA*) gene | Gene derived from *Synechocystis* sp. PCC 6803 (UniProt accession: P73833) and codon-optimized for recombinant expression in *E. coli* |
| 3 | Expression plasmid (pETDuet-1-*cphA*) | Recombinant plasmid containing the codon-optimized *cphA* gene cloned into the pETDuet-1 vector under the control of the T7 promoter and lac operator system |
| 4 | Expression vector (pETDuet-1) | Contains T7 promoter, lac operator, ribosome binding site (RBS), ampicillin resistance marker (AmpR), and f1 origin for recombinant protein expression in *E. coli* |
| 5  6 | Induction system  pLQ171_pETDuet-1-*cphA*-EGFP | Lactose used as both carbon source and inducer during recombinant CphA expression and CGP production  pETDuet-1-based recombinant plasmid encoding the CphA-EGFP fusion protein; construct size, 8,802 bp (~8.8 kb); expected molecular weight of the CphA-EGFP fusion protein, approximately 124.2 kDa; used for Western blot analysis of recombinant protein expression |
